# Novel acyclic nucleoside analogues as inhibitors of HIV-1 RT

**DOI:** 10.1186/1471-2334-12-S1-P43

**Published:** 2012-05-04

**Authors:** Anuradha Singh, Madhu Yadav, Dipti Yadav, Garima Kumari, Ramendra K Singh

**Affiliations:** 1 Nucleic Acids Research Laboratory, Department of Chemistry, University of Allahabad, Allahabad (UP)-211002, India

## Background

Nucleoside reverse transcriptase inhibitors (NRTIs) were the first drugs introduced for treatment of human immunodeficiency virus-1 (HIV-1) infection. These NRTIs may be cyclic or acyclic analogs of natural nucleosides. Both these analogs interact at active site on HIV-RT and compete with indigenous nucleosides/nucleotides, and thus, divert enzyme activity in manmade direction. All NRTIs follow three phosphorylation steps that convert the parent compound successively to 5'-triphosphate. These 5'-triphosphates act as alternate substrate for HIV-RT, and lead to chain termination when incorporated into the DNA chain as they don’t provide the 3'-OH function.

## Method

Development of acyclic allylic nucleoside analogs, which act as NRTIs against HIV, involves both the computational and synthetic methods. Designing is done keeping the Lipinski’s Rule of Five in focus and SAR studies were performed using DS 3.0 software. The ADMET descriptor and TOPKAT protocol available in DS 3.0 were used to predict these properties. The Lipinski’s Rule of Five was also used to determine the biological activity or druglikeness of the designed inhibitors.

## Result

All acyclouracil analogues formed 3-10 bonds with amino acids constituting the dNTP site on HIV-RT. The amino acids that interact with these molecules are Gln44, Lys46, Lys65, Arg72, Asp110, Asp113, Gln151, Asp185, Pro217, His221, Lys223 through H-bonding and π-π interaction.

## Conclusion

On the basis of SAR studies, acyclic allylic analogs of uracil bearing carbonyl and sulphonyl groups at N-3 position are expected to be probable lead molecules against HIV-RT. Biological screening is under process.

